# 

**Published:** 1858-12

**Authors:** 


☞ Prof. Flint left Buffalo in the early part of November to enter on the duties of his Chair in the New Orleans School of Medicine. He expects to return in the latter part of February next. His correspondents, in the meantime, will please direct their letters to him at New Orleans.

